# A novel roadmap connecting the ^1^H-MRS total choline resonance to all hallmarks of cancer following targeted therapy

**DOI:** 10.1186/s41747-020-00192-z

**Published:** 2021-01-15

**Authors:** Egidio Iorio, Franca Podo, Martin O. Leach, Jason Koutcher, Francis G. Blankenberg, Joseph F. Norfray

**Affiliations:** 1 High Resolution NMR Unit-Core Facilities, Istituto Superiore di Sanità, Viale Regina Elena, 299, 00161 Roma, Italy; 2 MRI Unit, Royal Marsden Hospital, Downs Road, Sutton, Surrey SM2 5PT UK; 3 Department of Medicine, Memorial Sloan Kettering Cancer Center, New York, NY 10065 USA; 4 Stanford University/MIPS, 725 Welch Road, Room #1860, Palo Alto, CA 94304 USA; 5 Emeritus, Chicago Northside MRI Center, 2818 N. Sheridan Rd, Chicago, IL 60657 USA

**Keywords:** Biomarkers, Choline, Magnetic resonance spectroscopy, Neoplasms, Unfolded protein response

## Abstract

This review describes a cellular adaptive stress signalling roadmap connecting the ^1^H magnetic resonance spectroscopy (MRS) total choline peak at 3.2 ppm (tCho) to cancer response after targeted therapy (TT). Recent research on cell signalling, tCho metabolism, and TT of cancer has been retrospectively re-examined. Signalling research describes how the unfolded protein response (UPR), a major stress signalling network, transduces, regulates, and rewires the total membrane turnover in different cancer hallmarks after a TT stress. In particular, the UPR signalling maintains or increases total membrane turnover in all pro-survival hallmarks, whilst dramatically decreases turnover during apoptosis, a pro-death hallmark. Recent research depicts the TT-induced stress as a crucial event responsible for interrupting UPR pro-survival pathways, leading to an UPR-mediated cell death. The ^1^H-MRS tCho resonance represents the total mobile precursors and products during the enzymatic modification of phosphatidylcholine membrane abundance. The tCho profile represents a biomarker that noninvasively monitors TT-induced enzymatic changes in total membrane turnover in a wide variety of existing and new anticancer treatments targeting specific layers of the UPR signalling network. Our overview strongly suggests further evaluating and validating the ^1^H-MRS tCho peak as a powerful noninvasive imaging biomarker of cancer response in TT clinical trials.

## Key points

- The ^1^H magnetic resonance spectroscopy total choline peak at 3.2 ppm (tCho), an imaging biomarker of membrane metabolism, is a signature of malignancy that monitors the biological response of the adaptive stress signalling network to anticancer therapy.
- The unfolded protein response (UPR), a major adaptive stress signalling network, modulates, rewires, and reprogrammes the cancerous “-omics”, thus modifying the total cell membrane turnover in response to a therapeutic stress.
- The UPR signalling works to maintain or increase tCho in tumours, whilst decreases in tCho occur during treatment-induced apoptosis.
- The tCho peak represents a potential noninvasive therapeutic biomarker that monitors UPR signalling-driven changes in total membrane turnover after molecular targeted therapy.

*Deciphering of the molecular mechanism of the “unfolded protein response” provides a wonderful example of how serendipity can shape scientific discovery* [1]

Peter Walter, 2009 Award Essay, American Society of Cell Biology

## Background

The goal of precision medicine is to accurately select and match targeted therapy (TT) with therapeutic biomarkers to reduce morbidity, increase survival and manage costs [2]. Currently, selection of a TT requires the identification of genetic mutations and expression patterns driving cancerous cellular reprogramming within a patient’s phenome [3]. Computer analysis of vast data sets, however, often fails to find a unifying therapeutic biomarker connecting complex gene activity in all cancerous cellular programmes in a given patient [4]. In addition, the heterogeneity of gene expression throughout primary tumour and secondary metastasis frequently results in the identification of multiple therapeutic biomarkers within the patient’s cancerous phenome further complicating treatment planning [2].

The ^1^H magnetic resonance spectroscopy (MRS) total choline peak (tCho) at 3.2 ppm may hold promise as a universal therapeutic imaging biomarker of TT-induced changes, as it directly reflects the regulation and modification of the membranous machinery sustained by integrated stress, genomic, proteomic and phenomic signalling. This review connects recent advances in cell signalling, ^1^H-MRS tCho detection and TT research, deciphering the major links amongst them to create a roadmap for better understanding and testing tCho as a universal biomarker of TT efficacy. We also highlight the potential clinical impact of tCho-based MRS imaging, along with its advantages compared with current biomarkers, and avenues for testing.

## Criteria adopted for reviewing

Cell signalling, tCho detection, and TT research were retrospectively re-examined and linked. Only peer-reviewed original research articles, reviews and molecular cellular biology textbooks were re-examined. Initially, the re-examination suffered by assuming tCho metabolism as the only key to interpret the observed fluctuations of this spectroscopic parameter. Only after acquiring a deeper understanding of how cell signalling transmits, regulates, modifies, and integrates the genetic code through the stress, genomic, proteomic, and phenomic layers of the unfolded protein response (UPR) signalling network, did a clearer mechanism of TT-induced tCho changes emerge.

The search engine for tCho connections to cell signalling and TT was initially the Index Medicus and later the Internet. To exclude the possibility of overlooking important signalling connections, references within original research articles, reviews, and molecular cell biology textbooks were cross-referenced against those referenced in this review. Confirmatory evidence from cell signalling and TT research identified a signalling mechanism orchestrating changes in the tCho resonance after TT. The authors regret limiting the selection of noteworthy research to those revealing a roadmap connecting tCho to an intracellular stress signalling network regulating total membrane turnover in all remaining, reprogrammed hallmarks after targeted therapies.

## Links amongst stress signalling, tCho and TT

### Cell signalling research re-examined

Recent signalling research describes a cellular adaptive stress signalling network that focuses on the genomic, proteomic and phenomic signalling. The significance of this adaptive stress signalling in the regulation of membrane turnover has so far been underappreciated. This review provides the background research supporting tCho turnover providing a biomarker that monitors UPR-regulated enzymatic modifications of membrane abundance.

**Fig. 1:**
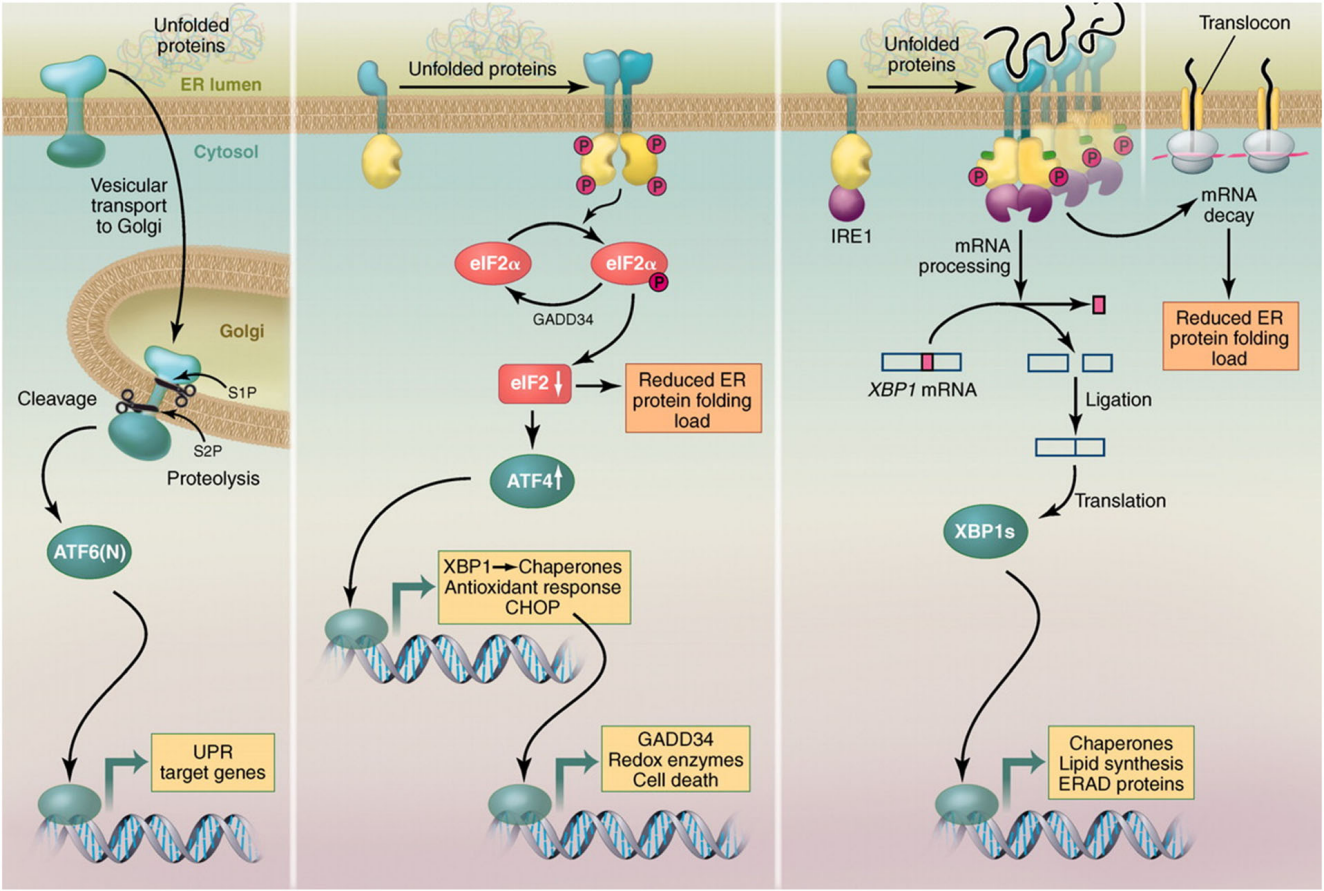
ATF6, PERK, and IRE1 branches of a layered UPR signalling network. Layer 1—unfolded proteins in the ER lumen. Layer 2—ATF6, PERK, and IRE1 transmembrane ER sensors. Layer 3—core processors. Layer 4—ATF6(N), ATF4, and XBP1s transcription factors. Layer 5—UPR genome. Layer 6—cellular programmes regulating cell death, lipid synthesis, ERAD, production of chaperones, redox enzymes, and GADD34. *ATF4*, Activated transcription factor 4; *ATF6*, Activating transcription factor 6; *ATF6(N)*, Activated transcription factor 6 N-terminal cytosolic fragment; *elF2*, Eukaryocyte initiation factor 2; *ER*, Endoplasmic reticulum, *ERAD*, Endoplasmic reticulum assisted degradation; *GADD34*, Growth arrest and DNA damage-inducible protein; *IRE1*, Inositol requiring enzyme 1; *P*, Phosphorylation; *PERK*, Double-stranded RNA-activated protein kinase-like ER kinase; *S1P*, Site-1 protease; *S2P*, Site-2 protease; *UPR*, Unfolded protein response; *XBP1s*, X-box binding protein1s with subscript s indicating nonconventional mRNA splicing transmits UPR signalling. Adapted with permission from Walter et al. [15]

In 1988, signalling researchers discovered a distinct set of cellular adaptations to stress now known as the UPR [5]. UPR is activated by accumulation of unfolded proteins in the endoplasmic reticulum (ER) membranous lumen and induces the synthesis of two ER chaperones, the glucose-regulated proteins GRP78 and GRP94 [5]. Between 1996 and 2011, Peter Walter’s team of UPR signalling researchers found three distinct signalling branches in this cellular layered network [1, 6–15] which are illustrated in Fig. 1 [15]. Their research documented a tight balance between the abundance of unfolded secretory proteins and the abundance of secretory membranes [7]. They also described UPR’s signal transduction and regulation of apoptosis and autophagy cellular programmes [10, 15].

Other UPR researchers between 2004 and 2012 described the mechanism of the inositol requiring enzyme 1 (IRE1) and activating transcription factor 6 (ATF6) signalling branches regulating the synthesis of ER membranes [16–20]. They documented that a rapid recycling of cytidylyltransferase may eliminate the need for its increased gene expression during membrane synthesis [17].

In 2015, a separate group of researchers connected the upstream layer which integrates multiple extrinsic and intrinsic cellular stress sensing pathways with the UPR signalling network. They also characterised the downstream cellular programmes as either pro-survival (including autophagy) or pro-death (apoptosis) [21–26]. More importantly, their articles provide a glimpse of UPR signalling connection to Hanahan’s hallmarks of cancer. Douglas Hanahan and Robert Weinberg coined the term hallmarks of cancer to organise and unravel the complexities of cellular programmes driving cancer [27]. Cancer alters cellular programmes to sustain proliferation, amplify growth, reprogramme metabolism, activate metastasis, resist cell death, induce angiogenesis, evade the immune system, facilitate invasion, enable inflammation, and create the tumour microenvironment [27, 28]. UPR signalling characterises autophagy as a pro-survival cellular programme [21–26] and corrects Hanahan’s misconception of autophagy as a chimeric pro-survival and pro-death cellular programme [27, 28]. UPR signalling also transduces and regulates apoptosis, a pro-death cellular programme (see Fig. 1) illustrating how a tumour alters, adjusts, and reprogrammes Hanahan’s hallmarks of cancer [15, 21–26]. UPR signalling expands Hanahan’s hallmarks to include a pro-survival hallmark (autophagy) and a pro-death hallmark (apoptosis) [21–26]. Research in UPR signalling fulfils the prediction by Hanahan and Weinberg of simplifying the complexities of cancer by discovering their signalling mechanisms [27].

UPR signalling is a signalling layered network as outlined by Gerhard Krauss and Peter Walter (Fig. 1) [15, 29]. In the *stress layer*, intrinsic and extrinsic cellular stress sensing pathways are converted and channelled into ER stress by their generation of unfolded secretory proteins in the ER membranous lumen [22]. Binding of co-factors and posttranslational modifications alter the response of the three ER stress sensors [26]. Interaction between, and activation and silencing of, UPR transcription factors create pleomorphic transcription factors that reset gene activity [21]. In the *genomic layer*, UPR also reprogrammes gene activity by regulating gene expression utilising microRNA to either degrade messenger ribonucleic acid (mRNA) or prevent mRNA from being translated [13, 21]. In the *proteomic layer*, modular, multi-protein complexes create different secretory signalling proteins allowing rapid and variable intracellular and intercellular signalling. Protein signalling also occurs through posttranslation modifications (phosphorylation, methylation, acetylation, oxidation, nitrosylation, ubiquitination, sumoylation) that increase domain interactions, allosteric configurations and effector input signalling [29]. In the *phenomic layer*, UPR rewires the pro-survival programmes to overcome ER stress, or if the ER stress is too excessive or too prolonged, the UPR orchestrates apoptosis, a pro-death programme [15, 21, 24, 26].

Central to a layer network is integration of signalling by core signalling processors. For UPR signalling, these include kinases (protein kinase A/cyclic adenosine, known as PKA/cAMP; protein kinase B/mammalian target of rapamycin, known as PKB/mTOR; and protein kinase C/diacylglycerol, known as PKC/DAG), switches (Ras GTPase/guanosine diphosphate/guanosine triphosphate, known as Ras/GDP/GTP, and death receptor 5, DR-5), cascades (mitogen-activated protein kinase, known as MAPK) and adaptors (proto-oncogene tyrosine-protein kinase Src, and growth factor receptor-bound protein 2, known as Grb2) [30–32]. UPR’s core signalling transducers increase the speed and flexibility in UPR signalling [30, 33]. Furthermore, the UPR signalling network is a complete set of biologic circuits employing both negative and positive feedback loops (see Fig. 1) [15, 30].

UPR signalling tightly controls and coordinates the secretory membranous machinery within the pro-survival and pro-death signalling pathways [15]. The classical secretory intracellular membranous compartments translate, fold, assemble (endoplasmic reticulum), modify (Golgi apparatus), transport (vesicles), store (vesicles), and secrete (vesicles) secretory proteins. Other non-classical secretory intracellular membranous compartments generate energy (mitochondria), remove oxidants (peroxisomes), sequester cytoplasmic unfolded secretory proteins (autophagosomes), recycle receptors (endosomes), shed receptors and cytokines (exosomes), and degrade unfolded secretory proteins and membranes (lysosomes) [34]. These membranous compartments are intracellular membranes (ICMs) and form the membranous machinery of the UPR.

The UPR signalling adjusts secretory protein synthesis, modification and trafficking to overcome fluctuations in intrinsic and extrinsic cellular stress [22, 26]. Secretory proteins reveal essential signalling components in cancer molecular oncology [33]. Secretory proteins comprise growth factors, receptors, cytokines, chemokines, extracellular matrix proteins, proteases, major histocompatibility complexes, and immunoglobulins. Secretory proteins are essential signalling components within the UPR signalling layered network. Cancer uses its secretory proteins to transform normal neighbouring cells into cancer-associated fibroblasts, tumour-associated macrophages, and cancer-associated endothelial cells. The transformed cells, in turn, use their secretome to reinforce the pro-survival hallmarks [33]. The cancer-associated fibroblasts promote tumour invasion with matrix-metalloproteinases 2 and 9, known as MMP2 and MMP9, and the hepatocyte growth factor known as HGF. The tumour-associated macrophages generate inflammation with the interleukin 1b; the cancer-associated endothelial cells’ secretome increases angiogenesis with the vascular endothelial growth factor [33]. Examples of other secretory proteins include serine/threonine-protein kinase B-Raf, serine/threonine-protein kinase C-Raf, anaplastic lymphoma kinase receptor, hepatocyte growth factor receptor, epidermal growth factor receptor, and cyclin-dependent kinase 4. Properly folded secretory proteins are essential signalling components in all pro-survival hallmarks and one pro-death hallmark [29].

#### UPR signalling in pro-survival hallmarks

**Fig. 2:**
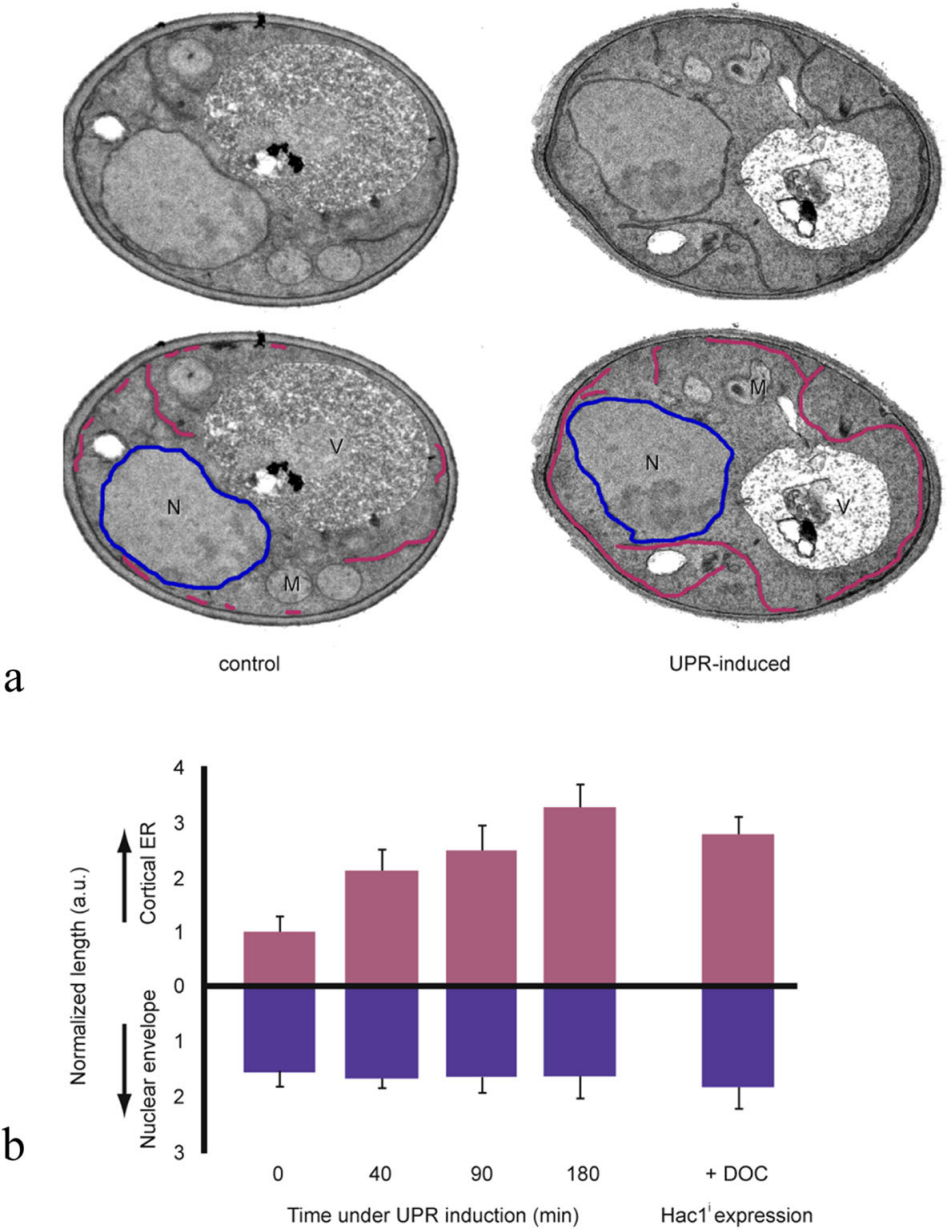
ER elongation after UPR induced stress. **a** Determination of ER elongation in cells before (control) and after UPR stress (UPR-induced). TEM examinations of thin sections of a control and an UPR-induced cell were magnified to a resolution of 140 nm (upper images) in which the cortical ER was outlined in magenta, and the nuclear envelope outlined in blue (lower images). Cursory glance reveals UPR stress causes ER elongation with inward displacement of the ER away from the plasma membrane. **b** Quantification of the cortical ER and nuclear envelope after UPR induction. Length of the ER (as traced in **a**) was measured and divided by area of the section generating a normalised length (a.u.). Data plotted relative to time 0. Measurement for each time point corresponds to mean of 25 independent cells. *ER*, Endoplasmic reticulum; *TEM*, Transmission electron microscopy; *UPR*, Unfolded protein response. Adapted with permission from Bernales et al. [10]

All UPR pro-survival signalling mechanisms maintain or increase membrane synthesis. The UPR pro-survival signalling utilises two transmembrane sensors, IRE1 and the activating transcription factor 6, ATF6 (see Fig. 1) [15]. Pro-survival signalling generates two corresponding transcription factors, X-box binding protein XBP1s with “s” indicating a product of spliced mRNA and ATF6(N) with N indicating N-terminal cytosolic fragment. Pro-survival transcription factors cause gene activation of phospholipid biosynthesis and membrane biogenesis (see Fig. 1) [21, 22]. UPR orchestrates early ER membrane synthesis and is documented by transmission electron microscopy as a fivefold increase in ER volume and a threefold increase in ER elongation after unfolded protein induced stress (Fig. 2) [10]. Figure 2 shows that staining after application of an ER immunofluorescent probe correlates with ER transmission electron microscopy findings. The ER probe is a sensitive reproducible tool that documents ER expansion [14]. Increase in ER volume reduces aggregation of misfolded proteins by providing adequate space for protein folding during glycosylation and disulphide bond formation [10]. Increase in ER elongation is also a source for autophagosome and lysosome membranes needed in autophagy [10, 34–36]. Secretory membrane synthesis is required in all pro-survival responses (proliferation, growth, angiogenesis, autophagy, metastasis, metabolism, invasion, inflammation, and immunotolerance).

**Fig. 3:**
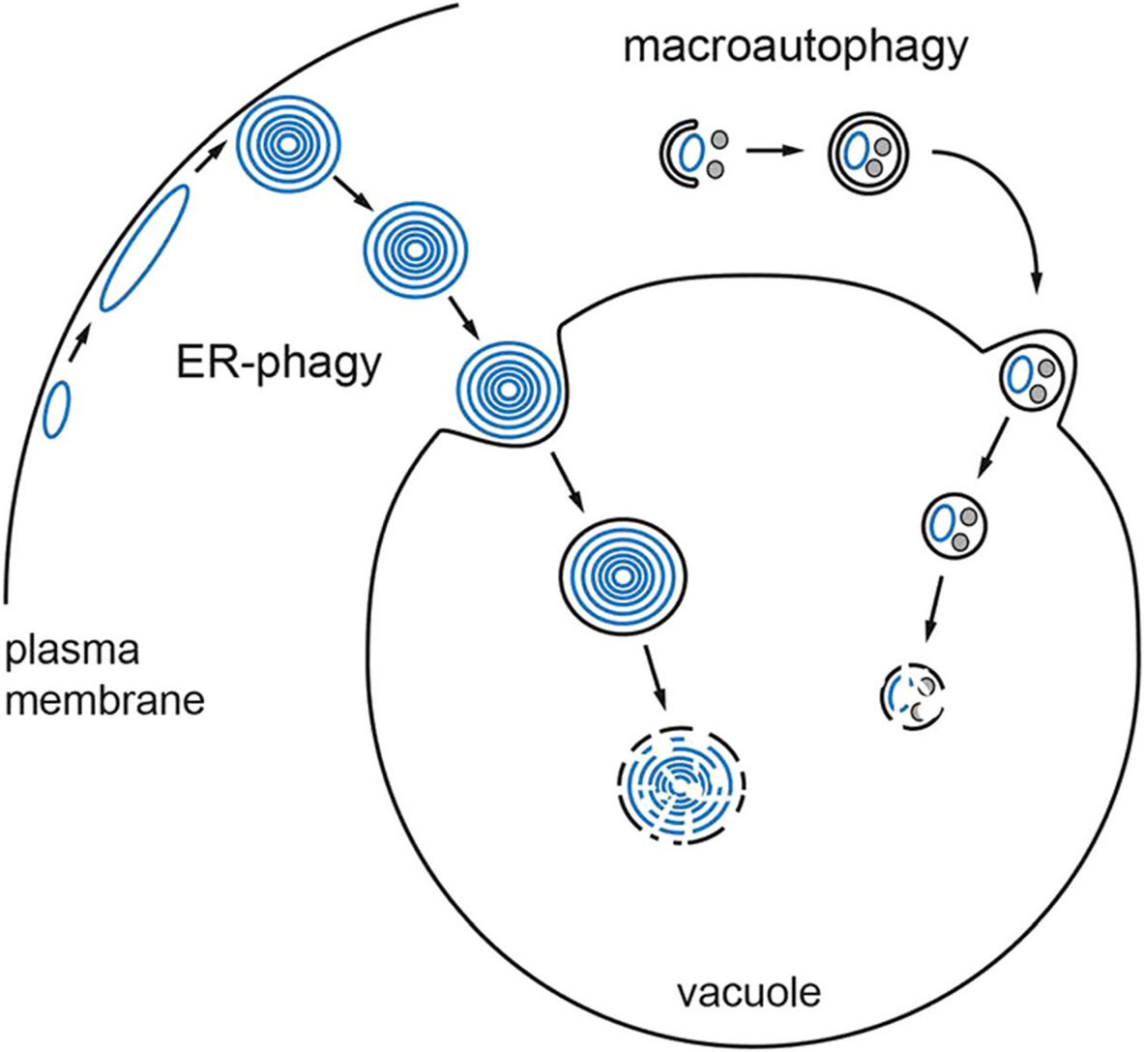
Pro-survival ER-phagy and macroautophagy degradation of cellular components in a vacuole to maintain cellular homeostasis. ER-phagy packages excessive ER abundance in ER-whorls (concentric blue circles) and degrades them within an intracellular membranous vacuole, the lysosome. Macroautophagy engulfs cytoplasmic, misfolded proteins (small, grey circles), and non-functioning, aged intracellular membranes (blue ovals) and degrades them within the lysosome. To maintain cellular homeostasis, the degraded products are recycled (not shown). *ER*, Endoplasmic reticulum. Reprinted with permission from Schuck et al. [38]

UPR pro-survival signalling also initiates autophagy degradation and recycling of catabolic products. Limited amounts of unfolded secretory proteins in the ER induce UPR genes for proteasome ER-associated degradation that spares the ICM [8]. Excessive amounts of unfolded secretory proteins in the ER overwhelm the proteasomes and activate the third ER transmembrane sensor, the double-stranded RNA-activated protein kinase-like ER kinase (PERK) (Fig. 1) [15, 21, 22]. PERK increases translation of two important autophagy transcription factors, the activating transcription factor 4 (ATF4) and the transcription factor C/EBP homologous protein, that activate three ER stress autophagy genes [37]. Autophagy genes are implicated in the formation of autophagosome membranes [37]. Autophagosomes envelope and package aggregated cytoplasmic proteins and by-stander ICM for bulk macroautophagy lysosomal degradation (Fig. 3) [37]. ER stress also induces ER-phagy, a distinct type of autophagy, where excessive ER membrane whorls invaginate directly into the lysosome for degradation. ER-phagy bypasses the autophagosome (Fig. 3) [38]. Lysosomal proteases degrade the misfolded proteins. Lysosomal phospholipases degrade the autophagosomes, by-stander cargo ICM and ER-whorls. Macroautophagy and ER-phagy are pro-survival hallmarks that degrade and recycle their products to maintain cellular nutrients, energy and homeostasis [37, 38].

#### UPR signalling in a pro-death hallmark

Excessive and/or prolonged intrinsic and extrinsic stresses initiate UPR apoptosis by generating excessive and/or prolonged accumulation of unfolded secretory proteins [12, 22, 25, 39]. Hanahan’s pro-survival cellular programmes suggested the existence of a pro-death cellular programme. The pro-death cellular programme was confirmed by research in 2014 and 2018 that identified a novel UPR signalling switch that regulates cell survival based on the degree of cellular stress [40, 41]. The death receptor DR-5 is a UPR protein switch regulating cell survival and cell death depending on the amount of ER stress. Two ER sensors, PERK and IRE1 (see Fig. 1), regulate the synthesis and degradation of DR-5 mRNA [15]. During an early and limited ER stress, IRE1 signalling predominates and degradation of DR-5 mRNA provides a window for adaptation by autophagy or resistance. During prolonged or excessive stress, PERK signal predominates and excessive synthesis of DR-5 combines with caspase-8 to drive a ligand-independent activation of the extrinsic pathway of apoptosis [40, 41]. The UPR PERK signal regulation of the extrinsic pathway of apoptosis is essential in immune induced programmed cell death [25, 39, 42]. The UPR signalling also regulates the intrinsic mitochondrial pathway of apoptosis by regulating the induction of pro-apoptotic proteins-Bcl-2-like protein 4, Bcl-2-like protein 11, RNA-binding protein Nova 1, and Bcl-2-binding component 3 [22, 39]. The UPR’s pro-death hallmark is apoptosis.

### tCho research re-examined

A review of investigations on detection and molecular significance in cancer of the ^1^H-MRS tCho peak centred at about 3.2 ppm documents this spectral resonance as a valid probe of membrane phospholipid metabolism [43–50].

#### The ^1^H-MRS tCho peak profile in cancer

**Fig. 4:**
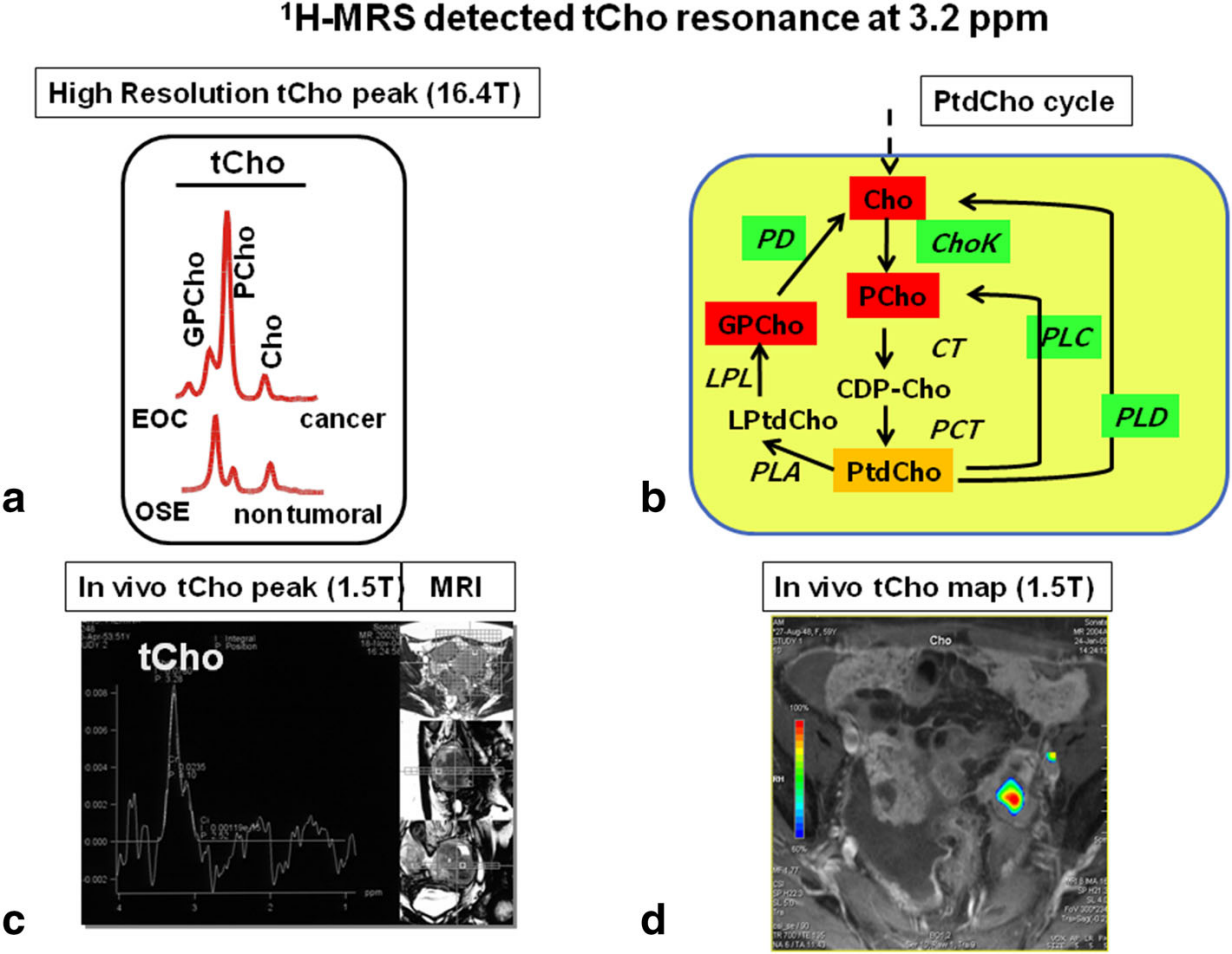
*In vitro* ^1^H-MRS detection of total choline (tCho) metabolic profile in epithelial ovarian cancer (EOC) cells and *in vivo* MRS/MRS imaging clinical examinations of an EOC patient. **a** High-resolution ^1^H-MRS tCho profile of aqueous extracts of ovarian surface epithelial (OSE) and epithelial ovarian cancer (EOC) cells. **b** Scheme of phosphatidylcholine metabolism. **c** *In vivo* single-voxel ^1^H-MRS tCho peak in a EOC patient. **d** *In vivo* ^1^H-MRSI tCho map in the same EOC patient as in **c**. Details in references [44–46, 49]. Enzymes: *ChoK*, Choline kinase (EC 2.7.1.32); *CT*, Cytidylyltransferase (EC 2.7.7.15); *LPL*, Lysophospholipase (EC 3.1.1.5); *PCT*, Phosphocholine transferase (EC 2.7.8.2); *PD*, Glycerophosphocholine phosphodiesterase (EC 3.1.4.2); *PLA*, Phospholipase A1 (EC 3.1.1.32); *PLA2*, Phospholipase A2 (EC 3.1.1.4); *PLC*, Phosphatidylcholine-specific phospholipase C (EC 3.1.4.3); *PLD*, Phospholipase D (EC 3.1.4.4). Metabolites: *CDP-Cho*, Cytidine diphosphate-choline; *Cho*, Free choline; *GPCho*, Glycerophosphocholine; *LPA*, Lysophosphatidate; *LPtdCho*, Lysophosphatidylcholine; *PCho*, Phosphocholine; *PtdCho*, Phosphatidylcholine; *tCho*, Total choline-containing metabolites (GPCho + PCho + Cho). Adapted with permission from Podo et al. [49]

The tCho resonance mainly arises from the nine protons of the trimethylammonium headgroups –N^+^(CH_3_)_3_ of major mobile choline-containing phospholipid metabolites (Fig. 4a), notably phosphocholine (PCho), glycerophosphocholine (GPCho), and free choline. These metabolites act both as precursors in the synthesis and derivatives in the catabolic pathways of the metabolic-functional phosphatidylcholine (PtdCho) cycle (scheme in Fig. 4b), whose activation is closely controlled by over-expression of cell receptors and oncogenes responsible for cell signalling deregulation in cancer cells [47–50]. PtdCho is the most abundant phospholipid of intracellular and extracellular membranes in eukaryotic cells. Typical features of the high-resolution ^1^H-MRS tCho resonance profile detected in cancer cells compared to that of nontumoural counterparts are a remarkable elevation of the PCho signal and adjustment from low to high values of the PCho/GPCho peak intensity ratio (see Fig. 4a). For these reasons, the ^1^H-MRS tCho profile has been identified as a metabolic signature of malignancy [44–51]. The enhanced PCho production in cancer cells is currently attributed to upregulation of choline kinase alpha [44–51]. Recent studies on breast and ovarian cancer cells documented up to 50% of the intracellular PCho pool can also derive from PtdCho-specific phospholipase C (PC-PLC) activity [49]. Although the identification of individual tCho components is practically lost at the lower spectral resolution allowed by current *in vivo* ^1^H-MRS equipment, the increase in PCho in cancer lesions typically produces a remarkable overall increase in the tCho peak. This allows for the tCho-based discrimination of tumoural from adjacent nontumoural tissues in single-voxel ^1^H-MRS as well as in multi-voxel ^1^H-MRS imaging in a routine clinical setting (examples in Fig. 4c, d) [44–46, 48, 49]. Recently, attention has been turned to focus on the question of whether the tCho resonance could also act as a therapeutic biomarker for monitoring changes in total membrane turnover before, during, and after TT [47–51].

#### Clinical relevance of *in vivo* tCho quantification

*In vitro* high-resolution ^1^H-MRS analyses of cell extracts allowed quantification of tCho concentration ([tCho]) in a variety of human cancer cells. For instance, a pioneering study by Eric Aboagye and Zaver Bhujwalla in 1999 [52] reported absolute [tCho] levels ranging from 0.5 to 4.7 mM and [PCho] levels ranging from 0.5 to 3.2 mM in breast cancer cell lines of different phenotypes, compared with nontumoural mammary epithelial cells ([tCho] 0.05–0.3 mM; [PCho] 0.0–0.1 mM). The same team of investigators also reported that different cell lines derived from primary or metastatic prostatic tumours had [tCho] levels between 1.0 and 5.1 mM, the [PCho] levels ranging from 0.5 to 2.4 mM, compared with the much lower values in nontumoural epithelial or stromal prostate cells ([tCho] 0.2–0.5 mM; [PCho] 0.1–0.4 mM) [53]; Egidio Iorio et al. reported [tCho] levels ranging between 5.2 and 8.5 mM, with [PCho] ranging between 4.0 and 7.0 mM in epithelial ovarian cancer cell lines, compared with significantly lower levels in nontumoural counterparts ([tCho] 2.0–2.5 mM; [PCho] 1.0–1.2 mM) [44, 45].

In substantial agreement with *in vitro* analyses of breast cancer cell lines, early *in vivo* single-voxel ^1^H-MRS examinations of breast cancer patients showed higher [tCho] levels in invasive ductal carcinoma (mean concentration 2.2 mM, range 0.0–8.5 mM) *versus* benign fibrosis and hyperplasia (mean value 0.2 mM, range 0.0–1.1 mM) [54].

An *in vivo* single-voxel water- and fat-suppressed ^1^H-MRS study on 48 patients at 1.5 T showed that the tCho peak integral (measured in arbitrary units and either expressed as absolute values or values normalised for the volume of interest) acted as a good marker of malignancy in breast cancer diagnosis [55] with high levels of diagnostic performance, both in terms of receiver operating characteristic analyses (area under the curve 0.917 or 0.941) and in terms of sensitivity (0.895 or 0.842) and specificity (0.923 or 0.885). Notably, a ^1^H-MRS study by the same team at 3.0 T showed that the absolute tCho concentration was kept at low levels (from 0.4 to 0.9 mM) in fertile young women over the menstrual cycle and independently of the use of oral contraceptives [56].

A recent ^1^H-MRS study at 1.5 T on 103 patients showed the potential of quantitative tCho evaluation to diagnose malignancy and lymph node status in suspicious breast lesions identified by multiparametric MRI [57]. At receiver operating characteristic analyses for prediction of malignancy, the area under the curve was 0.816 and 0.809 according to two independent readers (R1 and R2) with a cutoff of 0.8 mM tCho concentration to diagnose malignancy with a sensitivity of over 0.95. For prediction of lymph node metastasis, tCho measurements yielded an area under the curve of 0.760 (R1) and 0.788 (R2). At tCho levels < 2.4 mM, no metastatic lymph nodes were found. These results supported the potential of using ^1^H-MRS tCho quantification to downgrade suspicious multiparametric MRI-detected lesions and stratify the risk of lymph node metastasis for improving patient management.

The integration of quantitative ^1^H-MRS with other multiparametric MRI examinations at 3.0 T of patients with brain metastases from breast cancer, treated with a combination of bevacizumab (on day 1) with chemotherapeutic agents (on days 2–4) in 21-day cycles, indicated that the relative changes (Δ) in the [tCho/N-acetylaspartate] and [tCho/Creatine] peak ratios measured at the end of the therapy cycle correlated with the central nervous system-specific progression-free survival and overall survival [58]. These results supported the view that quantification of tCho-based ^1^H-MRS signatures may contribute, in combination with other multiparametric MRI biomarkers, to a better prediction of the survival outcome in patients with brain metastases from breast cancer.

It should also be underlined that *in vivo* single-voxel ^1^H-MRS and multi-voxel ^1^H-MRS imaging tCho quantification in a clinical setting is challenging because of stringent instrumental requirements, such as the need for high or very high magnetic field, “artefact-free” performance, robust water- and fat-suppression, accurate and reproducible spatial localisation [46]. Furthermore, despite continuous advances in technology, an inherent limitation in the quantification and interpretation of the *in vivo* ^1^H-MRS tCho peak derives from the narrow separation (within about 0.1 ppm) amongst the resonance frequencies of the trimethylammonium groups of individual metabolites contributing to the tCho profile, making it hard to measure relevant parameters such as PCho concentration and PCho/GPCho ratio. ^31^P-MRS allows however a much wider separation (about 3.5 ppm) amongst the resonance frequencies of the phosphomonoester and phosphodiester compounds contributing to the tCho molecular profile, thus allowing their quantification, although at the cost of a lower sensitivity of ^31^P-MRS *versus* ^1^H-MRS [59]. A recent study performed at 7 T on volunteers, combining water- and fat-suppressed ^1^H-MRS and adiabatic multi-echo ^31^P-MRS imaging, allowed separate estimates of [PCho] (0.1 mM) and [GPC] (0.1 mM), along with the concentration of phosphoethanolamine (≤ 0.2 mM), another phosphomonoester contributing with two protons to the tCho spectroscopic profile. These results suggested the potential use of a combination of *in vivo* ^31^P- and ^1^H-MRS at very high field to monitor quantitative changes in phospholipid metabolites in breast cancer lesions of patients treated with neoadjuvant chemotherapy, as successfully tested in a very recent study reported by the same research team [60].

#### The ^1^H-MRS tCho resonance as a potential therapeutic biomarker

Early testing of tCho as a potential therapeutic molecular biomarker suggested a general clinical impact [61, 62]. However, further testing of this spectroscopic parameter in different TT-treated tumours showed some unpredicted, puzzling fluctuations in tCho when compared to classical, dimensional biomarkers of tumour size and proliferation. The tCho peak typically decreases early compared to subsequent decreases in tumour size after selected chemotherapies [47]. Furthermore, the tCho peak could even paradoxically increase under conditions of decreased cell density induced by some TTs [47, 48, 63]. These puzzling changes in tCho peak intensity can be explained by considering that a direct comparison of this tumour’s molecular characteristics with changes in tumour size or cell density is not always justified. The tCho molecular biomarker in fact measures total membrane turnover from all ICMs and all extracellular membranes (ECMs) [50, 51]. The dimensional biomarkers of growth (size) and proliferation (cell density) measure instead only the visible surface area of the ECM and fail to measure the invisible surface area of the ICM within the cell. A review of molecular cell biology documents that the ICM and ECM differ in membrane abundance and kinetics (synthesis/degradation). A tenet in molecular cell biology documents that the total ICM equals about 90% of cell membrane surface area, whilst the total ECM equals the remaining 10% [34, 35]. During membrane synthesis, ICMs are generated first, with the ECM budding-off from pre-existing ICMs [36]; in contrast, during apoptotic membrane degradation, the caspases degrade the ICM before the macrophages degrade the ECM [64]. UPR signalling research supports the view that the “paradoxical” early increase in tCho represents autophagy. Massive synthesis of the ICM in surviving cells combined with ICM and ECM degradation of apoptotic cells creates a “paradoxical” increase in tCho despite a decrease in cell density [10]. The initially perceived weakness of tCho as a therapeutic molecular biomarker might therefore actually represent its hidden advantages over dimensional and other biological biomarkers.

#### The tCho profile monitors the total membrane turnover in all cancer hallmarks

As proposed above, the changes detected in the tCho peak area in response to a TT may not only reflect modifications directly induced by cancer therapy on oncogene-driven activation or deactivation of enzymes involved in the PtdCho cycle, but may also be effectively controlled and regulated by the UPR signalling network. The mechanism of the variable turnover of free choline, PCho and GPCho components of the tCho profile is in fact coordinated and integrated by UPR gene expression of metabolic enzymes during the synthesis and degradation of PtdCho membranes [8, 16–20].

As outlined in a 1992 review on basic principles of MRS of tumours, Martin Leach, Laurence Le Moyec and Franca Podo clarified that *in vivo* ^1^H-MRS protocols mainly detect at 3.2 ppm the signals from highly mobile aqueous (cytoplasmic) choline-containing phospholipid metabolites (*i.e.*, PCho, GPCho, and free choline) rather than the headgroups of membrane-bound choline-containing phospholipids, made invisible by the line-broadening induced by restricted molecular tumbling and segmental flexibility [65]. It appears also likely that the overall ^1^H-MRS-detected pool of aqueous mobile choline-containing metabolites is proportional to the total amount of membrane-bound choline-containing phospholipids, the two biochemical compartments being in continuous steady-state equilibrium, under control of the metabolic network responsible for phospholipid biosynthesis and catabolism [66]. Although advanced techniques of stoichiometric modelling of this metabolic network might help in the future in increasing our insights on exchanges between these two pools under different conditions of cancer progression and response to therapy, direct information on the existing proportionality factors between these two biochemical compartments is still lacking.

Despite the need for further investigations on this matter, the tCho resonance can be envisaged as a noninvasive molecular probe of total membrane turnover and can be proposed as a valuable biomarker for monitoring net changes in both ICM and ECM in all known UPR pro-survival hallmarks (proliferation, growth, angiogenesis, autophagy, invasion, inflammation, immunotolerance, metastasis, and metabolism) and in a UPR pro-death hallmark (apoptosis). In particular:

- *tCho increases* from enhanced membrane turnover of both ICM and ECM in all UPR pro-survival hallmarks. Increase in membrane turnover occurs early within 48–72 h in autophagy [63] and is delayed for weeks/months during the development of treatment resistance [62];
- *tCho decreases* from decreased membrane turnover of the ICM and ECM in a UPR pro-death hallmark. tCho decreases rapidly in 12–24 h in apoptosis [64] and slowly over days or weeks when autophagy fails to adequately recycle metabolites to cope with the therapeutic stress thereby switching on DR-5 elicited apoptosis [40, 41].

The puzzling tCho metabolic changes observed after some TTs, such as reported by Alissa Brandes et al. [67], may now be further explained by the activation of the UPR signalling which integrates total membrane turnover from both the ICM and ECM. Under these circumstances, the greatest fluctuations in the tCho therapeutic biomarker would directly reflect increases or decreases in the ICM, a set of membranes encompassing about 90% of the whole PtdCho within a cell.

### TT research re-examined

The promise of precision medicine is now being met by the rapidly expanding number of TTs [68]. They block the growth and spread of cancer by interrupting the cell signalling responsible for pro-survival cancer hallmarks, whilst creating an additional stress that may initiate apoptosis [39].

Pro-survival hallmarks are reprogrammed from the increase of intrinsic and/or extrinsic cellular stress that in turn increases the ER stress due to the build-up of unfolded secretory proteins [22, 24]. Intrinsic cellular stress signalling arising from cancerous mutations or loss of tumour suppressors increases the ER stress potentially overwhelming the ER folding capacity of the membranous machinery [22]. Extrinsic cellular stresses within the tumour microenvironment (loss of oxygen, nutrients, energy, or increase in reactive oxygen species, and addition of therapeutics), also increases ER stress by either (1) preventing the normal formation of intramolecular bonds or (2) the breakage of intramolecular bonds within secretory proteins [22, 25]. The UPR network converts and channels all the intrinsic and extrinsic stress signalling into ER stress. When ER stresses are too severe or prolonged, activation of a DR-5 protein switch initiates apoptosis, a pro-death hallmark [21–25].

The potency (and safety profile) of a TT determines its therapeutic efficacy [69]. TT potency correlates to the degree and duration of the therapeutic stress [69]. A severe therapeutic stress after a potent TT causes apoptosis with an early rapid decline in tCho in as little as 12–24 h [64–70]. A potent TT results in a rapid, prolonged decrease in tCho [69]. A less potent TT, with less therapeutic stress, demonstrates a relatively smaller decrease in tCho [70]. Blankenberg and Norfray document that a marked decrease in tCho correlates with the onset of apoptosis [64]. The levels of transformed stem, pericytes, endothelial, inflammatory and immune cells in the tumour microenvironment also contribute to treatment-induced decreases in tCho [28].

Responders show a decrease in tCho from cell death. Apoptosis is known to be a form of programmed cell death [70]. Targeted therapies may elicit both apoptosis and non-apoptotic cell death such as necrosis, mitotic catastrophe and senescence [71]. The tCho biomarker distinguishes between apoptosis (early decrease only in tCho) and necrosis (loss of all metabolites) [61]. The signature of non-apoptotic cell death in mitotic catastrophe and senescence is also a loss of cells reflected by a decrease in tCho. Synergistic TT combinations increase the therapeutic stress by preventing avenues of escape into multiple UPR pro-survival pathways and switch to a pro-death response.

Nonresponders show increases in tCho. Nonresponders utilise the UPR to evade therapeutic stress. The increase in tCho depends on UPR reprogramming of the remaining pro-survival pathways. An early increase in tCho within 48 h indicates autophagy from early UPR synthesis of ICM and recycling of degradation products of ICM and ECM [10]. A delay in the increase in tCho after several weeks or months indicates development of resistance from a tedious UPR reprogramming of the “-omics” in the remaining pro-survival hallmarks. Recent research confirms TT induces resistance by reprogramming and expanding the cancerous secretome within the remaining pro-survival hallmarks [72]. Turnover of the secretory membranous machinery within the remaining, reprogrammed pro-survival pathways explains why tCho increases during autophagy, resistance and competitive combination of TT agents. The value of tCho as a therapeutic biomarker in precision medicine is noninvasively distinguishing responders from nonresponders, thus allowing for timely personalised alterations in TT.

#### tCho as a biomarker in mono-TT research re-examined

Iorio and colleagues in the Podo’s team documented that tricyclodecan-9-yl-potassium xanthate, a competitive PC-PLC inhibitor, interrupts an essential enzymatic pathway in PtdCho degradation, thereby reducing by 30 to 40% the PCho pool available for PtdCho re-synthesis, local diacylglycerol production and phospholipid remodelling [45]. Follow-up research by the same team of investigators found that a D609-based TT blocks co-localisation of PC-PLC with the human epidermal growth factor receptor 2 (HER-2) resulting in HER-2 internalisation, loss of cell proliferation and decrease in mesenchymal traits [49]. The D609-based TT interrupts an enzymatic reaction chain contributing to cell signalling by blocking the PC-PLC enzyme from docking on HER-2 and forming a multi-protein signalling complex, thereby resulting in HER-2 ubitiquitination and internalisation [73].

Brandes et al. document that 17-N-allyamino-17-demethoxygeldanamycin, an inhibitor of the HSP90 ER chaperone, causes an apparently paradoxical increase in tCho at 48 h [67]. This HSP90-inhibitor inhibits the normal, folding of client secretory signalling proteins (HER-3, and the serine/threonine-protein kinases known as B-Raf, C-Raf, AKT) causing ER stress by increasing the accumulation of unfolded secretory proteins [22]. The accumulation of unfolded secretory proteins activates UPR autophagy. Recent TT research now provides the clinician with the options to personalise treatment. Increasing the dose of 17-N-allyamino-17-demethoxygeldanamycin [70], changing to a more potent HSP90 inhibitor, the 8-[(6-iodo-1, 3-benzodioxol-5-yl) sulfanyl]-9-[3-(propan-2-ylamino) propyl] purin-6-amine [69] or adding a synergistic combination of TT agents [69, 74] increases the therapeutic stress.

Heisoog Kim et al. document that another mono-TT, 4-[4-fluoro-2-methyl-1H-indol-5yl)oxy]-6-methoxy-7-3- (pyrrolidin-1-ylpropoxy) quinazoline, Cediranib, a pan vascular endothelial growth factor inhibitor, causes a complex therapeutic UPR stress response [75]. Between days 1 and 28 autophagy causes an early paradoxical increase in tCho. Between days 28 and 58, a prolonged stress causes apoptosis with a decrease in tCho. Between 58 and 128 days, resistance suggests invasive, epithelial-mesenchymal transformation with increase in tCho indicating treatment failure [76]. Experiments by Laura Abalsamo et al. in the Podo’ team also showed that PC-PLC inhibition blocked the epithelial-mesenchymal transformation in metastatic breast cancer cells [77] and could therefore exert synergistic effects in a combination TT on aggressive tumours.

#### tCho as a biomarker in combination TT research re-examined

Even though 17-N-allyamino-17-demethoxygeldanamycin is a mono-TT, its HSP90 inhibition has a variety of specific synergistic effects. HSP90 inhibition inactivates and degrades multiple client signalling proteins such as HER-2, the serine/threonine-protein kinases C-Raf and AKT, the epidermal growth factor receptor known as EGFR and the mast/stem cell growth factor receptor known as SCFR, all involved in multiple oncogenic pro-survival hallmarks [70].

## Present scenarios and future perspectives

There is a pressing need for a robust therapeutic molecular biomarker that monitors all cancer hallmarks after TT. Deciphering recent cell signalling research uncovers a UPR signalling roadmap connecting the therapeutic tCho biomarker of membrane turnover to all remaining, reprogrammed known hallmarks of cancer after a targeted therapeutic stress. The feasibility of a therapeutic biomarker and signalling roadmap simplifying complex signalling mechanisms after TT is documented by two recent clinical TT studies [67, 75]. Some questions still left open by current molecular interpretations based upon purely metabolic/enzymatic approaches may find answer by gaining further insights, as suggested by the present review, on the role of the stress UPR signalling network in rewiring total membrane turnover and therefore affecting ^1^H-MRS tCho profile in response to a given TT. In the frame of this proposed, more comprehensive interpretation, tCho could also identify and quantify autophagy, apoptosis, and resistance after TT. The UPR signalling network may also overcome the limitations of earlier cancer signalling maps [3, 27, 28]. The UPR roadmap in fact depicts an entire signalling circuit, places modifiers in their appropriate layers and identifies essential signalling targets.

The greatest potential impact of tCho imaging is the simplification of a treatment response assessment that can be clouded by complex variations occurring in stress, genomics, proteomics and cancer hallmarks in a given patient. tCho reflects the therapeutic stress as transmitted, regulated, modified and integrated through a UPR cellular adaptive stress signalling network. Monitoring tCho could in the future replace the assessment of multiple stress, genomic, proteomic, and phenomic biomarkers. The tCho peak could distinguish in quantitative terms between UPR pro-survival signalling (increase in tCho) and UPR pro-death signalling (decrease in tCho) after TT [75]. The tCho peak could also distinguish between the pro-survival hallmarks of autophagy (rapid, early increase in tCho within 12 to 24 h) and resistance (slow, delayed increase in tCho within weeks to months) after TT [75]. The tCho peak could be potentially used to quantify the effectiveness of TT in eliciting a pro-death response with an alternative TT potentially showing a faster and more prolonged return of tCho down to the levels observed in normal tissues. Monitoring tCho could also be a potential robust biomarker of multidrug efficacy with antagonistic combinations resulting in an increase and synergistic combinations demonstrating a decrease in tCho [72]. The tCho peak could also determine the optimal time for alterations in the therapy. A rapid, early increase in tCho from autophagy could represent an indication for increasing the therapeutic dose, increasing the therapeutic frequency or changing to a synergistic combination of antitumour agents to possibly elicit apoptosis [69, 70, 74]. A slow, delayed increase in tCho due to the development of resistance may also indicate that a biopsy is necessary to discover new genomic targets.

The tCho biomarker has several inherent strengths. The tCho biomarker fulfils the criteria of a relevant biomarker [57]; tCho monitors membrane turnover of the mobile choline-containing phospholipid metabolites during UPR-regulated/integrated enzymatic modification across all hallmarks of cancer in the cancerous phenotype after TT; tCho is a direct endogenous biomarker of turnover of mobile choline-containing phospholipid metabolites; tCho also indirectly reflects the pool of bound choline-containing phospholipids, that are in equilibrium with their mobile precursors and derivatives within the cellular membrane biomass. Monitoring of therapeutic responses in a noninvasive manner allows a longitudinal monitoring to guide therapeutic decisions. The tCho detection does not require radiation or injection of contrast. The tCho peak is quantifiable (although in terms of the sum of three major metabolites of the PtdCho cycle, in which PCho is often the predominant component) and reproducible for clinical therapeutic trials and pharmacological research at multisite and at high-field strengths and is supported by multiple manufacturers. The tCho peak centred at 3.2 ppm is also highly specific. Sensitivity of tCho ^1^H-MRS continues to improve in clinical and preclinical MR imaging with faster acquisitions, increased coil sensitivity and higher field magnets. In clinical units, multi-voxel ^1^H-MRS acquisitions interrogate the core of the tumour, as well as the periphery allowing autocrine and paracrine signalling to be monitored in different regions/micro-environments of the tumour and possibly residual and recruited cancerous cells [78]. The tCho peak centred at 3.2 ppm arises from all major mobile choline phospholipid metabolites with low overlapping with (or low contributions from) other MRS peaks and does not require subtler peak assignment with the assistance of a metabolomics spectral database [78]. Use of tCho as a biomarker may also overcome the problem that current UPR therapeutic biomarkers monitor only specific mechanisms and pathways [5, 79].

The use of tCho as a biomarker has several weaknesses including the low sensitivity of clinical ^1^H-MRS equipment and lack of automation. Low sensitivity arises from low signal-to-noise ratio and increased noise in the electronic signalling chain. Advancements in high-field magnets/coils, software acquisitions, and computer processing continue to improve the sensitivity of tCho detection. Recently, artificial intelligence has been shown to boost sensitivity by improving the signal-to-noise ratio, reducing noise from motion, and generating faster images (spectra). Artificial intelligence replaces the conventional reconstruction chain with a data driven reconstruction between the sensor domain and the image (spectrum) domain [80]. The key to future automation is further progress in software data processing.

This review lays the groundwork for scientific testing of a ^1^H-MRS molecular therapeutic biomarker of total membrane turnover in all hallmarks of cancer after targeted therapy. Adding tCho to ongoing clinical trials of TT and comparison with other clinical trial biomarkers would test the tCho biomarker robustness. Adding tCho to an ongoing trial would rapidly generate “evidence-based” proof of tCho impact on diagnostic accuracy, therapeutic decisions, patient outcome and cost to society. In the USA, a search of National Institutes of Health funding of 2019 molecular cancer biomarkers indicates that this institution is receptive to funding a molecular therapeutic choline biomarker [81, 82]. Other avenues of potential funding include current National Cancer Institute, Cancer Imaging Program initiatives and Radiology Society of North America opportunities [83].

During the final revisions to our review, some preclinical research articles suggested targeting essential components in cancer’s amplified pro-survival hallmarks. Martin Leach’s team suggested targeting the critical role of cytidylyltransferase enzyme in autophagosome membrane synthesis to block autophagy, a pro-survival hallmark [84]. Jason Koutcher’s team suggested targeting the macrophage colony stimulating factor 1 receptor in an effort to block immunotolerance, a pro-survival hallmark [85].

In conclusion, we are strongly convinced that a deeper understanding of cellular adaptation to stress reveals that ^1^H-MRS-detected tCho, a marker of membrane turnover, directly reflects and monitors all known hallmarks of cancer after TT. This vision offers a perspective to be verified by clinical studies.

## Data Availability

Not applicable

## Abbreviations

DR: Death receptor
ECM: Extracellular membrane
ER: Endoplasmic reticulum
GPCho: Glycerophosphocholine
HER: Human epidermal growth factor receptor
HSP90: Heat shock protein 90 kDa
ICM: Intracellular membrane
MRI: Magnetic resonance imaging
mRNA: Messenger ribonucleic acid
MRS: Magnetic resonance spectroscopy
PCho: Phosphocholine
PC-PLC: Phosphatidylcholine-specific phospholipase C
PERK: Double-stranded RNA-activated protein kinase-like ER kinase
PtdCho: Phosphatidylcholine
tCho: Total choline
TT: Targeted therapy
UPR: Unfolded protein response
